# Co-Inoculation with *Staphylococcus equorum* and *Lactobacillus sakei* Reduces Vasoactive Biogenic Amines in Traditional Dry-Cured Sausages

**DOI:** 10.3390/ijerph18137100

**Published:** 2021-07-02

**Authors:** Igor Dias, Marta Laranjo, Maria Eduarda Potes, Ana Cristina Agulheiro-Santos, Sara Ricardo-Rodrigues, Ana Rita Fialho, Joana Véstia, Maria J. Fraqueza, Margarida Oliveira, Miguel Elias

**Affiliations:** 1 MED—Mediterranean Institute for Agriculture, Environment and Development, IIFA-Instituto deInvestigação e Formação Avançada, Universidade de Évora, Pólo da Mitra, Ap. 94, 7006-554 Évora, Portugal; mlaranjo@uevora.pt (M.L.); mep@uevora.pt (M.E.P.); acsantos@uevora.pt (A.C.A.-S.); sirr@uevora.pt (S.R.-R.); rita.b.fialho@gmail.com (A.R.F.); joana.vestia@gmail.com (J.V.); elias@uevora.pt (M.E.); 2 CIEQV—Life Quality Research Centre, Avenida Dr. Mário Soares n 110, 2040-413 Rio Maior, Portugal; margarida.oliveira@esa.ipsantarem.pt; 3 ESAS, UIIPS—Instituto Politécnico de Santarém, Quinta do Galinheiro, S. Pedro, 1001-904 Santarém, Portugal; 4 Departamento de Medicina Veterinária, Escola de Ciências e Tecnologia, Universidade de Évora, Pólo da Mitra, Ap. 94, 7006-554 Évora, Portugal; 5 Departamento de Fitotecnia, Escola de Ciências e Tecnologia, Universidade de Évora, Pólo da Mitra, Ap. 94, 7006-554 Évora, Portugal; 6 CIISA—Centro de Investigação Interdisciplinar em Sanidade Animal, Faculdade de Medicina Veterinária, Universidade de Lisboa, Avenida da Universidade Técnica, 1300-477 Lisboa, Portugal; mjoaofraqueza@fmv.ulisboa.pt; 7 LEAF—Linking Landscape, Environment, Agriculture and Food, Instituto Superior de Agronomia, Universidade de Lisboa, Tapada da Ajuda, 1349-017 Lisboa, Portugal

**Keywords:** dry-cured sausages, starter cultures, staphylococci, lactic acid bacteria, food safety, biogenic amines, *Listeria monocytogenes*, food quality

## Abstract

Dry-cured sausages are traditional in Mediterranean countries, and Paio do Alentejo (PA) is one of the most popular in South Portugal. The aim of the present work was to evaluate the effect of combined starters on the safety and quality of PA preserving its sensory quality. Physicochemical parameters, namely pH and water activity (a_W_), microbiological parameters, biogenic amines, color, texture, and sensory attributes were assessed. Three starter cultures were used, namely *Staphylococcus equorum* S2M7 and *Lactobacillus sakei* CV3C2, both separate and combined with the 2RB4 yeast strain at a concentration of 10^6^ cfu/g. Dextrose 0.25% was added to the meat batter. Starters had a significant effect on the reduction of a_W_ values (0.845 to 0.823). The treatment with *L. sakei* as well as the co-inoculation of *L. sakei* with *S. equorum* effectively reduced the *L. monocytogenes* counts to undetectable levels. Sausages co-inoculated with *S. equorum* S2M7/*L. sakei* CV3C2 showed a significant reduction in the content of vasoactive amines, namely tryptamine (26.21 to 15.70) and β-phenylethylamine (4.80 to 3.69). Regarding texture, control PA showed higher hardness values, and the starters promoted the cohesiveness of the batter while reducing chewiness. The studied starters did not compromise the sensory characteristics of PA.

## 1. Introduction

Dry-cured sausages are traditional food products that are greatly diverse in terms of raw materials, organoleptic characteristics, and manufacturing methods. Paio do Alentejo is a popular dry-cured sausage in Portugal because it is manufactured using pork meat from autochthonous breeds as well as typical nonmeat ingredients in small processing units according to traditional practices specific to each geographical area. This type of sausage has long been spontaneously fermented using empirical methods, but sometimes, the sensory characteristics of the final products vary. In Portugal, the use of starters has not been a common practice in micro and small processing units [1]. However, manufacturing units are becoming more interested in the use of starter cultures in the production of fermented sausages, due to their potential improvement in safety and in standardizing the desirable technological properties [2,3,4]. These starter cultures should be autochthonous, i.e., isolated from the native microbiota of these products, so that they will be better adapted to the specific environmental conditions [5].

In fermented meat products, bacteria including lactic acid bacteria (LAB) and Gram-positive catalase-positive cocci (G^+^C^+^C), such as coagulase-negative staphylococci (CNS), but also yeasts and molds influence the technological properties of the product and its quality and safety [3,6]. Therefore, the most frequently used starters in the meat processing industry belong to the four cited microbial groups.

LAB ferment sugars thus boosting the production of lactic acid. The consequent reduction in pH reduces the growth rate of undesirable microorganisms [7,8]. However, and given the fact that LAB are among the most competitive microorganisms throughout the manufacturing process, they are considered to be biopreservatives and bioprotectors. In fact, together with intrinsic food factors, such as pH, temperature and a_W_, they can impair the growth of pathogenic and spoilage microorganisms, making food products safer even without the use of conservation techniques, such as modified atmospheres, high pressure treatments, and chemical or other preservatives [9].

CNS are able to reduce nitrate and degrade hydrogen peroxide, with advantages at the quality and color stability level, and metabolize nitrogenous and lipid compounds, improving flavor [10,11]. According to Cocconcelli and Fontana [12], CNS have the ability to release enzymes, lipases, and proteases capable of forming low molecular weight compounds, such as peptides, amino acids, aldehydes, amines, and fatty acids that influence texture and the development of aroma compounds.

Yeasts and molds are used less frequently as starter cultures. The application of molds and yeasts as surface starter cultures, normally by immersion or spraying, can improve specific sensory and external characteristics [13]. Surface starters form a protective layer, which favors color formation and hinders the occurrence of premature autoxidation phenomena of fats due to the activity of catalase [14].

Biogenic amines (BA) are nitrogenous compounds of low molecular weight formed from amino acids by decarboxylation or from aldehydes and ketones by amination and transamination [15]. The most prevalent biogenic amines in meat and meat products are tyramine, putrescine, cadaverine, and histamine [16,17]. Formation of BA depends on the availability of specific amino acids, the presence of bacteria with decarboxylase activity, and on the establishment of conditions favorable to bacterial growth and enzymatic activity [4]. It should be noted that BA are thermostable, as further steps do not eliminate them [18] and could contribute to the formation of nitrosamines with the nitrite derivatives [19,20]. Despite some studies that have reported the inefficiency of starters in reducing the content of BA [21,22], recent works have shown that autochthonous starter cultures may control the accumulation of BA in fermented meat products [1,23,24].

The aim of the present study was to evaluate the effects of different autochthonous starter cultures used both in separate and in mixed cultures on the safety and quality of Paio do Alentejo, a traditional Portuguese sausage manufactured on a small scale in a local manufacturing unit in the Alentejo region. Moreover, starters were used to help in the control of an existing problem with *Listeria monocytogenes* in the manufacturing unit, together with other corrective and preventive measures.

## 2. Materials and Methods

### 2.1. Dry-Cured Sausage Manufacturing and Sampling

Paio do Alentejo, a traditional dry-cured sausage, was manufactured in a local factory using commercial black pig breed (Alentejano pig breed × Duroc pig breed) meat.

Pork meat trimmings (70% lean meat/30% fat) were mechanically cut into cubes of approximately 25 mm and mixed with white wine (8.0% *v*/*v*), salt (2.5% *w*/*w*), red pepper (*Capsicum annuum* L.) paste (2.5% *w*/*w*), garlic (*Allium sativum* L.) paste (0.8% *w*/*w*), polyphosphates (0.06% *w*/*w*), nitrates (0.007% *w*/*w*), nitrites (0.003% *w*/*w*), ascorbic acid (0.03% *w*/*w*), and sodium ascorbate (0.02% *w*/*w*). A total of 150 kg of meat batter was prepared and then divided into five portions of 30 kg each. Five treatments were considered: 1—control (no starter cultures added); 2—*Staphylococcus equorum* S2M7; 3—*Lactobacillus sakei* CV3C2; 4—*S*. *equorum* S2M7/*L*. *sakei* CV3C2; and 5—*S*. *equorum* S2M7/*L*. *sakei* CV3C2/yeast 2RB4.

*Staphylococcus* starter strains were selected for their performance in the phenotypic characterization tests, namely nitrate reductase, lipolytic and proteolytic activities, as well as absence of resistance to antimicrobials and decarboxylase activity [25]. *Lactobacillus* starter strains were selected for their bacteriocinogenic profile and the absence of both resistance to antimicrobials and decarboxylase activity [25,26].

Starter culture composition and concentrations were selected based on previous trials [27] and were inoculated in the meat batter. All cultures were inoculated to achieve a final concentration of each starter strain of 10^6^ cfu/g of meat batter.

Three independent manufacturing batches of each treatment were prepared. Food grade dextrose (0.25%) was added to all treatments.

Seasoned and inoculated meat batter was stored under controlled conditions at 5 °C and 90% relative humidity (RH) for 72 h and then stuffed into desalted pork natural casings 50 to 55 mm in diameter. Sausages were smoked for 24 h at 18.0 to 24.0 °C and 28.0–72.0% RH in a traditional smokehouse. After smoking, drying was carried out in a controlled storeroom at 8.0–12.0 °C and at an RH between 60–80% for approximately 30 days 38–40% initial weight loss was reached.

Two sausages per treatment and per batch were analyzed throughout the curing process at three different steps: meat batter (immediately before stuffing), half-cured sausage (10 days after stuffing), and end-product (38–40% weight loss).

pH, a_W_, microbiological parameters, and contents of biogenic amines were determined at all curing steps. Color, texture profile and sensory analyses were performed only for end-products. Samples were immediately processed for physicochemical, microbiological, and sensory analyses and stored at −20 °C until analysis of the content of biogenic amines.

### 2.2. Physicochemical Analyses

#### 2.2.1. Determination of pH and a_W_

For the determination of pH and a_W_, samples were prepared and measurements were made as described previously [1], following ISO 2917 [28], for pH measurements. Five replicates per sample were used for both determinations.

#### 2.2.2. Color

Color CIELab chromatic coordinates were measured as described previously [1]. Five replicates per sample were examined.

#### 2.2.3. Texture Profile Analysis (TPA)

Texture profile analysis (TPA) was performed at room temperature (20 ± 1 °C) using a Stable Micro System TA-Hdi (Stable Micro Systems, Godalming, United Kingdom), as described previously [29,30]. Samples were prepared according to the procedures described by Dias et al. [1]. Five replicates per sample were analyzed.

### 2.3. Microbiological Analyses

Several microbiological parameters were analyzed following international standards and established procedures: mesophiles ISO 4833-1 [31]; psychrotrophic microorganisms ISO 17410 [32]; lactic acid bacteria ISO 15214 [33]; staphylococci [34]; yeasts and molds ISO 21527-2 [35]; enterobacteria ISO 21528-2 [36]; and *Listeria monocytogenes* ISO 11290-2 [37]. *Salmonella* spp. detection was performed with VIDAS (bioMérieux, Marcy-l’Étoile, France) and confirmed according to ISO 6579-1 [38] as described previously [34]. All microbiological analyses were performed in triplicate, and the results are expressed as log colony-forming units (cfu)/g, except for *L*. *monocytogenes* counts, which are reported as cfu/g.

### 2.4. Biogenic Amine Profiles

The content of biogenic amines was assessed as described previously [34,39]. Briefly, eight grams of each previously homogenized sample were extracted with 0.4 M perchloric acid aqueous solution and filtered. 1,7-Diaminoheptane was used as internal standard. Biogenic amines were then derivatized with dansyl chloride under alkaline conditions. The extract was diluted in acetonitrile; filtered through an Acrodisc 25 mm GHP, GF 0.45 lm membrane (Gelman Sciences, Inc., Port Washington, NY, USA); and injected in-to an HPLC system (Thermo Scientific Dionex, Ultimate 3000, Waltham, MA, USA). Chromatographic conditions were as follows: A RP-18 reverse phase column (5 µm of 4.0 × 125 mm and 100 Å) was used (Merck, Kenilworth, NJ, USA), coupled to an Alliance Separation Module 2695 (Waters, Milford, MA, USA), along with a gradient elution program that combines aqueous ammonium acetate solution and ace-tonitrile (Panreac, Barcelona, Spain), and detection was conducted at 254 nm using a Dual k UV/Vis Detector 2487 (Waters, Milford, MA, USA).

All samples were extracted in duplicate; each replicate was twofold derivatized and injected in duplicate. Tryptamine, β-phenylethylamine, putrescine, cadaverine, histamine, tyramine, spermidine, and spermine were quantified and are expressed in mg/kg of fresh weight. The content of vasoactive amines was calculated, summing tryptamine, β-phenylethylamine, histamine, and tyramine [15]. The total content of biogenic amines was the sum of each individual amine. Chromatographic data were analyzed with Chromeleon software version 6.8 (Thermo Scientific Dionex, Waltham, MA, USA).

### 2.5. Sensory Analysis

Panelists were selected and trained according to ISO 8586-1 [40] in a sensory evaluation room prepared in accordance with ISO 8589-1 [41].

Thirty minutes prior to each session, sausages were sliced (3 mm thick) and slices randomly distributed in white dishes, each identified with a random three-digit number. Crackers and mineral water were supplied to the panelists as palate cleansers.

The sensory evaluation attributes studied were color intensity, off-colors, marbled appearance, aroma intensity, and off-aromas. The panelists were asked to evaluate these attributes using a quantitative descriptive analysis with a scale ranging from 0 to 100 corresponding to “no perception” or “maximum perception”. Due to the presence of *Salmonella* spp. in some samples, only a visual and olfactive sensory analysis was performed. Each of the 10 panelists evaluated six samples per session.

### 2.6. Statistical Analysis

Data were analyzed using STATISTICA v.12.0 software from Statsoft (StatSoft Inc, 1984–2014, Tulsa, OK, USA). Outliers were detected using the Grubbs test (α = 0.05). Factorial or one-way ANOVAs were performed, and significantly different means were compared with Tukey’s HSD test (*p* < 0.05).

## 3. Results

3.1. pH and a_W_

Table 1 summarizes the results for pH and a_W_ of sausages subjected to the different treatments throughout the curing process.

For pH, significantly different mean pH values were observed between meat batter and the other two curing steps (half-cured sausages and end-products). Regarding an evaluation by curing step, the sausages inoculated with *S*. *equorum* S2M7/*L*. *sakei* CV3C2 showed an initial mean value (5.29 ± 0.51) significantly lower than that of sausages with *L*. *sakei* CV3C2 (5.48 ± 0.31). Regarding end-products, sausages inoculated with *L*. *sakei* CV3C2 had the lowest mean pH value (4.94 ± 0.07), and the only pH mean value lower than that of the control (4.97 ± 0.14).

As for a_W_, significant differences were observed between curing steps, with significantly lower mean values for the end-products. Concerning meat batter, inoculated sausages generally showed lower a_W_ values. Regarding half-cured sausages, control sausages still had a significantly higher mean a_W_ (0.948 ± 0.007). Except for the end-products inoculated with *L*. *sakei* CV3C2 (0.852 ± 0.002), all other sausages presented a significant reduction in the a_W_ mean values when compared to the control (0.845 ± 0.024), thus contributing to their safety.

### 3.2. Characterization of the Microbiota of Sausages

Table 2 shows no differences between control and inoculated sausages for the same curing step. However, in end-products, inoculated sausages tended to have higher counts of mesophiles, psychrotrophic microorganisms, and LAB. Regarding staphylococci, the sausages inoculated with *S*. *equorum* S2M7/*L*. *sakei* CV3C2/yeast 2RB4 showed the highest number.

No significant differences were observed for enterobacteria between treatments; however, their mean values were significantly lower in end-products, probably associated with the increase in LAB and the consequently lower pH and the lower a_W_ values.

*L*. *monocytogenes* was present in all curing steps. When *L*. *sakei* CV3C2 was inoculated alone or combined with *S*. *equorum* S2M7, the elimination of *L*. *monocytogenes* was more effective, to levels below the legal limit of 100 cfu/g, according to regulation 2073/2005 [42].

*Salmonella* spp. were present throughout the curing process but absent in end-products, with the exception of sausages inoculated with *S*. *equorum* S2M7.

### 3.3. Biogenic Amines

Table 3 generally shows that the content of biogenic amines decreased throughout the curing process. Moreover, the content of biogenic amines of inoculated sausages was lower than that of control sausages throughout the entire process.

Natural polyamines, namely spermidine and spermine, did not show large variations in their mean values during the curing process. Cadaverine, putrescine, and tyramine were the most abundantly detected biogenic amines in end-products, in descending order.

The contents in histamine and tyramine reduced over time and were lower in end-products (13 and 114 mg/kg, respectively).

Sausages inoculated with *S*. *equorum* S2M7/*L*. *sakei* CV3C2/yeast 2RB4 had the highest mean values (148.12 ± 20.75 mg/kg), while the co-inoculation of *S*. *equorum* S2M7/*L*. *sakei* CV3C2 significantly reduced the content of vasoactive biogenic amines. Moreover, the total content of biogenic amines globally decreased during ripening, with higher contents in control end-product sausages (973.01 ± 140.14 mg/kg) and sausages inoculated with *S*. *equorum* S2M7/*L*. *sakei* CV3C2 (792.72 ± 175.93 mg/kg) showing significantly lower contents. Concerning end-products, all treatments showed a mean content of total biogenic amines below 1000 mg/kg.

### 3.4. Color

Table 4 summarizes the color data for each treatment. Regarding L*, significant differences were observed between treatments with the sausages co-inoculated with *S*. *equorum*/*L*. *sakei* CV3C2/yeast 2RB4 being the darkest. No significantly different results were obtained for all other color parameters.

### 3.5. Texture Profile Analysis (TPA)

The results for the texture profile analysis (TPA) are shown in Table 5. Hardness values tended to be higher in control sausages. Regarding cohesiveness and resilience, no statistical differences were observed between treatments. Nevertheless, sausages inoculated with *L*. *sakei* CV3C2 showed the highest values, which might indicate more cohesive meat batter. For chewiness, higher values were obtained in the control treatment, which indicates that the inoculated sausages were easier to chew.

### 3.6. Sensory Analysis

Regarding sensory analysis (Table 6), the panelists did not detect significant differences for any of the evaluated attributes. Nevertheless, *L*. *sakei* CV3C2 inoculated sausages presented the highest mean color intensity (74 ± 15) and lowest mean value for off colors (0 ± 1). Control sausages had a lower aroma intensity (67 ± 17), and those inoculated with *S*. *equorum* S2M7/*L*. *sakei* CV3C2/yeast 2RB4 the highest (74 ± 13).

## 4. Discussion

Paio do Alentejo is a traditionally manufactured dry-cured high-quality sausage with characteristic organoleptic features that however needs to meet all legal standardization and food safety criteria.

Although pH and a_W_ usually contribute to the stability of sausages [13], in this work, starters did not have a noticeable effect on the pH of sausages. The fact that starters were not able to significantly lower pH, compared to the control, indicates that the lactic microbiota naturally present in the meat batter (to which dextrose was provided) also exhibits a high acidifying ability. In fact, dextrose can be immediately metabolized by all LAB present in the meat batter, autochthonous and starters, as their main source of energy. Our pH values were lower than those of Elias et al. [43] for Paio do Alentejo inoculated with a commercial culture (TEXEL^®^ ELSE BR) of *Lactobacillus* spp., *Micrococcaceae*, and yeast and an experimental starter culture with *L*. *sakei*/*S*. *xylosus*, and those of Simion et al. [44] for traditional Romanian sausages (Dacia) inoculated with a mixed culture of *L*. *sakei* CECT5764 and *S*. *equorum* SA25. One possible reason for our lower pH values is the use of dextrose (0.25%).

Regarding a_W_, inoculated sausages generally showed lower values, therefore contributing to food safety. pH also contributes to the drying process, due to the decrease in the water holding capacity of meat proteins, when pH values reach the isoelectric point (5.0-5.2), with the consequent reduction in a_W_ [45,46]. Control sausages (0.845 ± 0.024) and sausages inoculated with *L*. *sakei* CV3C2 (0.852 ± 0.002) had significantly higher a_W_ values, probably because they had lower pH values when compared to the other treatments. Our a_W_ values are similar to those of Simion et al. [44] and lower than those of other authors [1,43,47].

Enterobacteria counts were 2.24–2.75 log cfu/g in end-product sausages, which are borderline values for ready-to-eat foods according to the Health Protection Agency guidelines (2–4 log cfu/g) [48]. However, similar results been reported previously for dry-fermented sausages from Portugal and other Mediterranean countries [25,29]. Nevertheless, these values are higher than those reported by other authors for Portuguese and Italian sausages, respectively [1,49], indicating the need to improve hygiene procedures and to use better quality raw materials.

In present study, *L*. *monocytogenes* was present in most analyzed samples. Other authors reported the presence of *L*. *monocytogenes* in inoculated and non-inoculated sausages, but this presence was drastically reduced throughout the curing process, in some cases, to values below the detection limit of the method [47,50]. However, Lebert et al. [51] confirmed the presence of *L*. *monocytogenes* in three of nine ready-to-eat sausages produced in France, with mean values between 1.2 and 2.8 log cfu/g, i.e., values sometimes higher than those obtained in the present study and exceeding the legal limit (100 cfu/g) [42].

*Salmonella* spp. were absent in end-products, except in those inoculated with *S*. *equorum* S2M7. Some outbreaks caused by *Salmonella* spp. have been identified in European fermented sausages, such as those reported by Gossner et al. [52] and Kuhn et al. [53] for a French sausage and a Danish salami, respectively. Biogenic amines levels generally decreased throughout the curing process. Although this is not always the case, other authors have reported a similar behavior [54]. On the contrary, Xie et al. [55] verified increases throughout the production process. Laranjo et al. [56] and Simion et al. [44] showed average values that did not follow the same trend for all amines, i.e., some contents increased, others decreased, and others increased until the intermediate stage of curing and decreased again in the finished product. These variations are likely associated with the manufacturing process as well as with the microbiota that has a major influence on the decarboxylation of amino acids, precursors of biogenic amines [13,20].

Dry-fermented sausages can easily accumulate high levels of BA, especially putrescine, cadaverine, and tyramine, the most abundant biogenic amines in the present study [16,17,57], probably due to the high numbers of enterobacteria, LAB, and staphylococci, the main bacterial groups responsible for the formation of BA [15].

Histamine and tyramine are the most toxic biogenic amines [58,59] and are consequently very relevant for food safety [60]. Nuñez et al. [61] reported that for healthy adults, foods containing more than 500 mg/kg histamine and 1000 mg/kg tyramine are considered toxic or likely to jeopardize consumer health. The concentrations of histamine (3.17 ± 2.20 to 12.96 ± 3.92 mg/kg) and tyramine (88.44 ± 21.49 to 113.99 ± 32.99 mg/kg) obtained in end-products in the present work were much lower than those indicated by [61], and the treatment with *S*. *equorum* S2M7*/L*. *sakei* CV3C*2* showed the lowest concentrations in all curing steps.

The co-inoculation of *S*. *equorum* S2M7 with *L*. *sakei* CV3C*2* promoted of 70% reduction in the histamine content when compared to the control sausages in end-products. Authors such as Wang et al. [62] and Casquete et al. [63] also observed pronounced reductions in the content of histamine in sausages inoculated with starter cultures.

For vasoactive amines, Papavergou et al. [64] suggest 200 mg/kg as an indicator of good manufacturing practices and safe consumption. In the present study we observed a reduction throughout the curing process, and in end-products all sausages showed values below 200 mg/kg. Nevertheless, sausages inoculated with *S*. *equorum* S2M7/*L*. *sakei* CV3C2 significantly had the lowest mean value, representing 28.65% fewer vasoactive amines (111.00 ± 23.66 mg/kg), than control sausages (155.58 ± 37.29 mg/kg). This corroborates the previous starter selection, which showed that *S*. *equorum* S2M7 and *L*. *sakei* CV3C2 were low producers of biogenic amines [26].

In general, inoculated sausages had lower concentrations of biogenic amines in end-products, except for the treatment with the yeast strain, which seemed to increase the levels of tryptamine and histamine. Higher contents in biogenic amines had been reported previously for sausages inoculated with *Debaryomyces* and *Candida* strains [65].

In the present work, no significant differences were observed between the different treatments regarding most color parameters, as had been reported previously by [62,66,67,68] contrary to the findings of Ravyts et al. [69] and Talon et al. [70], who reported the positive contribution of starter cultures to sausage color.

The fact that the control sausages were harder could be associated with some proteolytic action of starters that softened the inoculated sausages [71,72].

In general, we may conclude that inoculation with starters did not depreciate the sensory characteristics of the sausages as had been reported previously by others [44] and even seemed to have some positive effect, namely in terms of aroma intensity, which had also been reported by other authors [73,74].

## 5. Conclusions

The inoculation of Paio do Alentejo with starters did not have a noticeable effect on the pH or improve color. However, significantly lower a_W_ values were obtained for inoculated sausages, except for sausages inoculated with *L*. *sakei* CV3C2.

The absence of significant differences, particularly for LAB, staphylococci, and yeasts, between inoculated and control sausages could be explained by the fact that starters do not “add” to the established microbiota but rather replace it by competitive exclusion.

The co-inoculation of *S*. *equorum* S2M7 and *L*. *sakei* CV3C2 promoted a reduction close to 30% and 20% respectively for vasoactive and total amines.

Regarding texture parameters, control sausages showed higher hardness values, and the use of starter cultures promoted the cohesiveness of meat batter and the reduction of chewiness.

In summary, the co-inoculation of Paio do Alentejo with *S*. *equorum* and *L*. *sakei* significantly reduced vasoactive biogenic amines. Moreover, the use of starter cultures did not compromise the quality of traditional dry-cured sausages regarding their sensory acceptability.

## Figures and Tables

**Table 1:** Effect of starter cultures on pH and a_W_ of sausages.

Parameters	Treatment	Curing Steps		
		Meat Batter	Half-Cured Sausage	End-Product
pH	1	5.48 **^A,ab^** ± 0.25	5.05 **^B,c^** ± 0.08	4.97 **^B,bc^** ± 0.14
	2	5.46 **^A,ab^** ± 0.28	5.20 **^B,a^** ± 0.09	5.05 **^B,ab^** ± 0.14
	3	5.48 **^A,a^** ± 0.31	5.13 **^B,b^** ± 0.09	4.94 **^B,c^** ± 0.07
	4	5.29 **^A,b^** ± 0.51	5.06 **^B,c^** ± 0,09	5.10 **^AB,a^** ± 0.01
	5	5.42 **^A,ab^** ± 0.32	5.19 **^B,a^** ± 0.09	5.10 **^B,a^** ± 0.10
a_W_	1	0.967 **^A,a^** ± 0.008	0.948 **^B,a^** ± 0.007	0.845 **^C,a^** ± 0.024
	2	0.962 **^A,bc^** ± 0.006	0.937 **^B,bc^** ± 0.009	0.826 **^C,b^** ± 0.031
	3	0.960 **^A,b^** ± 0.008	0.941 **^B,b^** ± 0.004	0.852 **^C,a^** ± 0.002
	4	0.960 **^A,b^** ± 0.007	0.941 **^B,b^** ± 0.004	0.823 **^C,b^** ± 0.030
	5	0.963 **^A,ab^** ± 0.002	0.934 **^B,c^** ± 0.004	0.824 **^C,b^** ± 0.014

Data are expressed as means ± SD. 1—Control; 2—*S*. *equorum* S2M7; 3—*L. sakei* CV3C2; 4—*S. equorum* S2M7/*L*. *sakei* CV3C2; 5—*S*. *equorum* S2M7/*L*. *sakei* CV3C2/yeast 2RB4. For the same treatment and in the same row, distinct capital letters (^A–C^) represent significantly different means (*p* < 0.05). For each curing step and in the same column, distinct lowercase letters (^a–c^) represent significantly different means (*p* < 0.05).

**Table 2:** Effect of starter cultures on microbiological parameters of sausages.

Parameters	Treatment	Curing Steps		
		Meat Batter	Half-Cured Sausage	End-Product
mesophiles	1	7.00 **^B^** ± 0.77	8.46 **^A^** ± 0.67	7.38 **^B^** ± 0.60
	2	7.23 **^B^** ± 0.96	7.70 **^A^** ± 0.65	7.65 **^AB^** ± 0.54
	3	7.16 ± 0.70	7.77 ± 0.29	8.39 ± 0.97
	4	7.72 ± 1.20	8.61 ± 0.82	8.03 ± 1.06
	5	7.35 ± 0.76	8.12 ± 1.07	8.48 ± 1.19
psychrotrophicmicroorganisms	1	6.60 ± 1.17	7.01 ± 1.22	5.66 ± 0.29
	2	6.81 **^A^** ± 0.99	6.50 **^AB^** ± 0.37	5.69 **^B^** ± 0.26
	3	6.74 ± 0.75	6.51 ± 0.58	6.20 ± 0.18
	4	7.32 ± 1.50	7.45 ± 1.29	5.89 ± 0.44
	5	7.18 ± 1.09	7.01 ± 1.22	6.48 ± 0.52
LAB	1	6.64 **^B^** ± 0.57	7.95 **^A^** ± 0.30	8.06 **^A^** ± 0.77
	2	6.58 **^B^** ± 0.39	7.59 **^A^** ± 0.28	7.96 **^A^** ± 0.67
	3	6.32 **^B^** ± 0.27	7.70 **^A^** ± 0.45	8.49 **^A^** ± 1.15
	4	6.80 **^B^** ± 0.60	7.93 **^AB^** ± 0.14	8.15 **^A^** ± 1.09
	5	7.01 **^B^** ± 0.26	8.18 **^AB^** ± 1.11	8.56 **^A^** ± 1.10
staphylococci	1	9.14 ± 0.66	10.17 ± 1.77	8.68 ± 1.03
	2	8.97 ± 1.42	9.26 ± 0.98	8.34 ± 0.49
	3	7.57 ± 1.48	9.14 ± 0.79	8.49 ± 0.71
	4	10.88 ± 3.96	10.47 ± 1.47	8.38 ± 2.14
	5	8.40 ± 1.74	9.66 ± 1.10	10.31 ± 1.29
enterobacteria	1	5.99 **^A^** ± 0.49	6.35 **^A^** ± 1.13	2.75 **^B^** ± 0.36
	2	6.55 **^A^** ± 1.16	5.54 **^A^** ± 0.24	2.69 **^B^** ± 0.50
	3	6.73 **^A^** ± 0.67	5.63 **^A^** ± 0.40	2.24 **^B^** ± 0.39
	4	7.02 **^A^** ± 1.34	6.45 **^A^** ± 1.04	2.48 **^B^** ± 0.40
	5	6.54 **^A^** ± 0.42	6.62 **^A^** ± 1.30	2.51 **^B^** ± 0.55
yeasts	1	3.88 ± 0.48	4.33 ± 1.09	4.61 ± 0.35
	2	3.78 **^B^** ± 0.23	3.15 **^C^** ± 0.18	4.70 ^A^ ± 0.47
	3	3.90 ± 0.90	3.82 ± 0.85	4.85 ± 0.42
	4	5.73 ± 2.44	4.56 ± 0.69	4.96 ± 0.74
	5	4.04 ± 0.37	4.47 ± 0.77	4.74 ± 0.69
molds	1	0.17 ± 0.41	0.67 ± 1.21	0.58 ± 1.20
	2	0.50 ± 0.84	0.25 ± 0.60	<DL
	3	<DL	<DL	0.33 ± 0.82
	4	<DL	<DL	<DL
	5	<DL	<DL	<DL
*L. monocytogenes*	1	2.22 ± 2.38	1.52 ± 1.91	2.06 ± 2.38
	2	2.00 ± 2.10	1.82 ± 1.91	2.17 ± 2.37
	3	2.12 ± 2.17	2.47 ± 2.73	<DL
	4	2.14 ± 2.22	2.26 ± 2.06	<DL
	5	2.50 ± 2.55	1.92 ± 1.87	2.17 ± 2.43
*Salmonella* spp.	1	present in 6/6 samples	present in 6/6 samples	ND
	2	present in 5/6 samples	present in 6/6 samples	present in 1/6 samples
	3	present in 1/6 samples	present in 2/6 samples	ND
	4	present in 5/6 samples	present in 4/6 samples	ND
	5	present in 3/6 samples	present in 3/6 samples	ND

Data are expressed as means ± SD. < DL: below the detection limit of the corresponding analytical method (10 cfu/g for molds and 100 cfu/g for *L*. *monocytogenes*). ND—Not detected (absence in 25 g). Results are expressed in log cfu/g. 1—Control; 2—*S*. *equorum* S2M7; 3—*L*. *sakei* CV3C2; 4—*S*. *equorum* S2M7/*L*. *sakei* CV3C2; 5—*S*. *equorum* S2M7/*L*. *sakei* CV3C2/yeast 2RB4. For the same treatment and in the same row, distinct capital letters (^A–C^) represent significantly different means (*p* < 0.05).

**Table 3:** Effect of starter cultures on the content of biogenic amines (mg/kg fresh weight) of sausages.

Parameters	Treatment	Curing Steps		
		Meat Batter	Half-Cured Sausage	End-Product
tryptamine	1	50.42 **^A,ab^** ± 5.78	38.46 **^B,ab^** ± 5.80	26.21 **^C,ab^** ± 5.59
	2	35.66 **^A,c^** ± 11.95	26.79 **^B,c^** ± 4.32	14.73 **^C,c^** ± 4.61
	3	43.44 **^A,bc^** ± 5.42	31.50 **^B,bc^** ± 5.40	19.28 **^C,bc^** ± 5.20
	4	40.32 **^A,bc^** ± 6.28	25.88 **^B,c^** ± 9.38	15.70 **^C,c^** ± 6.08
	5	59.61 **^A,a^** ± 16.64	47.60 **^AB,a^** ± 16.97	35.60 **^B,a^** ± 16.43
β-phenylethylamine	1	20.22 **^A^** ± 0.84	12.75 **^B,a^** ± 0.87	4.80 **^C,a^** ± 0.83
	2	17.63 **^A^** ± 5.50	11.75 **^B,ab^** ± 0.31	3.85 **^C,b^** ± 0.28
	3	19.36 **^A^** ± 0.72	11.90 **^B,ab^** ± 0.72	3.98 **^C,b^** ± 0.70
	4	19.08 **^A^** ± 0.56	10.77 **^B,b^** ± 3.09	3.69 **^C,b^** ± 0.51
	5	20.47 **^A^** ± 0.47	13.00 **^B,a^** ± 0.46	5.16 **^C,a^** ± 0.57
putrescine	1	466.47 **^A,a^** ± 51.42	401.79 **^B^** ± 51.69	329.11 **^C^** ± 50.82
	2	366.30 **^b^** ± 145.51	327.98 ± 91.38	255.70 ± 90.69
	3	407.36 **^A,ab^** ± 50.99	342.86 **^B^** ± 51.05	270.25 **^C^** ± 50.34
	4	422.68 **^A,ab^** ± 79.72	324.99 **^B^** ± 116.88	278.92 **^B^** ± 65.91
	5	417.69 **^A,ab^** ± 67.86	352.77 **^A^** ± 67.19	283.86 ^B^ ± 77.31
cadaverine	1	570.34 **^A^** ± 100.89	517.32 **^AB,a^** ± 101.11	439.42 **^C^** ± 98.35
	2	488.14 ± 157.62	483.83 **^ab^** ± 46.10	407.69 ± 47.15
	3	533.10 **^A^** ± 72.79	480.35 **^A,ab^** ± 73.51	403.29 **^B^** ± 71.73
	4	492.71 **^A^** ± 90.66	393.79 **^AB,b^** ± 128.61	353.27 **^B^** ± 49.91
	5	485.12 **^A^** ± 51.29	431.95 **^B,ab^** ± 51.66	360.81 ^C^ ± 89.05
histamine	1	32.81 **^A,ab^** ± 9.10	25.72 **^A.ab^** ± 9.03	10.58 **^B,ab^** ± 8.01
	2	29.54 **^A,ab^** ± 11.34	24.81 **^A,ab^** ± 6.72	10.13 **^B,ab^** ± 5.03
	3	30.99 **^A,ab^** ± 7.51	23.93 **^A.ab^** ± 7.46	8.20 **^B,ab^** ± 6.92
	4	26.50 **^A,b^** ± 2.24	18.06 **^B,b^** ± 5.57	3.17 **^C,b^** ± 2.20
	5	36.21 **^A,a^** ± 4.01	29.13 **^B,a^** ± 4.01	12.96 **^C,a^** ± 3.92
tyramine	1	162.13 **^A^** ± 33.62	139.50 **^AB^** ± 33.49	113.99 **^B^** ± 32.99
	2	141.65 ± 60.07	134.18 ± 41.76	108.80 ± 40.96
	3	137.87 **^A^** ± 17.37	115.31 **^B^** ± 17.25	89.72 **^C^** ± 16.95
	4	136.85 **^A^** ± 21.41	104.22 **^B^** ± 35.39	88.44 **^B^** ± 21.49
	5	142.85 **^A^** ± 19.95	120.22 **^B^** ± 20.06	94.40 **^C^** ± 19.52
spermidine	1	12.48 **^A^** ± 1.34	12.01 **^AB^** ± 1.33	11.02 **^B^** ± 1.38
	2	11.16 ± 3.71	11.79 ± 1.36	10.86 ± 1.34
	3	12.19 **^A^** ± 1.14	11.72 **^AB^** ± 1.12	10.78 **^C^** ± 1.12
	4	12.24 ± 0.73	10.83 ± 3.11	11.37 ± 0.94
	5	12.75 **^A^** ± 0.90	12.28 **^AB^** ± 0.90	10.82 **^b^** ± 0.78
spermine	1	46.81 ± 11.12	42.89 ± 11.06	37.88 ± 10.92
	2	40.71 ± 16.30	40.96 ± 10.39	35.97 ± 10.21
	3	43.28 ± 9.46	39.37 ± 9.39	34.40 ± 9.31
	4	44.26 **^A^** ± 6.05	36.77 **^B^** ± 11.58	38.16 **^AB^** ± 5.95
	5	47.17 **^A^** ± 6.16	43.24 **^AB^** ± 6.16	35.29 **^B^** ± 6.24
vasoactive amines	1	265.58 **^A^** ± 36.90	216.44 **^B,a^** ± 36.64	155.58 **^C,a^** ± 37.29
	2	224.48 **^A^** ± 80.05	197.53 **^A,ab^** ± 39.78	137.51 **^B,ab^** ± 39.95
	3	231.66 **^A^** ± 21.72	182.64 **^B,ab^** ± 21.41	121.18 **^C,ab^** ± 21.95
	4	222.75 **^A^** ± 22.37	158.93 **^B,b^** ± 48.96	111.00 **^C,b^** ± 23.66
	5	259.15 **^A^** ± 20.21	209.95 **^B,a^** ± 20.03	148.12 **^C,a^** ± 20.75
total amines	1	1361.68 **^A^** ± 141.42	1190.45 **^B,a^** ± 141.92	973.01 **^C,a^** ± 140.14
	2	1130.79 **^A^** ± 381.98	1062.09 **^AB,ab^** ± 146.00	847.73 **^C,ab^** ± 149.84
	3	1227.58 **^A^** ± 102.13	1056.95 **^B,ab^** ± 103.01	839.90 **^C,ab^** ± 101.51
	4	1194.64 **^A^** ± 177.13	925.30 **^A,b^** ± 295.33	792.72 **^B,b^** ± 175.93
	5	1221.88 **^A^** ± 95.62	1050.20 **^B,ab^** ± 94.67	838.90 **^C,ab^** ± 94.05

Data are expressed as means ± SD. 1—Control; 2—*S*. *equorum* S2M7; 3—*L*. *sakei* CV3C2; 4—*S*. *equorum* S2M7/*L*. *sakei* CV3C2; 5—*S*. *equorum* S2M7/*L*. *sakei* CV3C2/yeast 2RB4. For the same treatment and in the same row, distinct capital letters (^A–C^) represent significantly different means (*p* < 0.05). For each curing step and in the same column, distinct lowercase letters (^a–c^) represent significantly different means (*p* < 0.05).

**Table 4:** Effect of starter cultures on the color parameters of end-product sausages.

Treatment	Color Parameters				
	L * (Lightness)	a * (Redness/Greenness)	b * (Yellowness/Blueness)	C * (Chroma)	H° (Hue Angle)
1	42.32 **^a^** ± 4.63	18.58 ± 2.86	15.64 ± 5.00	24.44 ± 6.74	39.16 ± 6.74
2	43.41 **^a^** ± 5.03	19.43 ± 3.66	15.72 ± 5.26	25.14 ± 5.81	38.13 ± 5.91
3	41.32 **^ab^** ± 4.26	19.15 ± 3.80	15.87 ± 5.53	25.00 ± 6.17	38.69 ± 6.14
4	42.00 **^a^** ± 4.66	19.13 ± 2.97	16.26 ± 4.40	25.21 ± 4.79	39.80 ± 5.31
5	38.14 **^b^** ± 5.24	18.37 ± 2.61	15.02 ± 4.79	23.90 ± 4.62	38.55 ± 6.42

Data are expressed as means ± SD. 1—Control; 2—*S*. *equorum* S2M7; 3—*L*. *sakei* CV3C2; 4—*S*. *equorum* S2M7/*L*. *sakei* CV3C2; 5—*S*. *equorum* S2M7/*L*. *sakei* CV3C2/yeast 2RB4. In the same column, different letters (^a^ and ^b^) represent significantly different means (*p* < 0.05).

**Table 5:** Effect of starter cultures on TPA parameters of end-product sausages.

Treatment	Texture Parameters					
	Hardness (N)	Adhesiveness (N s^−1^)	Cohesiveness	Springiness	Resilience	Chewiness (N)
1	63.169 **^a^** ± 15.151	−3.398 ± 1.741	0.594 **^ab^** ± 0.035	0.881 ± 0.094	0.133 **^ab^** ± 0.014	33.325 **^a^** ± 10.504
2	49.606 **^c^** ± 10.171	−2.778 ± 1.529	0.600 **^ab^** ± 0.053	0.913 ± 0.097	0.134 **^ab^** ± 0.029	27.036 **^b^** ± 6.168
3	58.404 **^ab^** ± 14.308	−2.837 ± 1.852	0.622 **^a^** ± 0.058	0.901 ± 0.173	0.144 **^a^** ± 0.022	32.158 **^ab^** ± 8.002
4	52.785 **^bc^** ± 9.826	−3.003 ± 1.827	0.581 **^b^** ± 0.044	0.889 ± 0.070	0.128 **^b^** ± 0.025	27.192 **^b^** ± 5.355
5	51.220 **^bc^** ± 11.199	−2.629 ± 1.553	0.609 **^ab^** ± 0.046	0.966 ± 0.256	0.136 **^ab^** ± 0.016	29.777 **^ab^** ± 8.926

Data are expressed as means ± SD. 1—Control; 2—*S*. *equorum* S2M7; 3—*L*. *sakei* CV3C2; 4—*S*. *equorum* S2M7/*L*. *sakei* CV3C2; 5—*S*. *equorum* S2M7/*L*. *sakei* CV3C2/yeast 2RB4. In the same column, different letters (^a–c^) represent significantly different means (*p* < 0.05).

**Table 6:** Effect of starter cultures on the sensory attributes of sausages evaluated in end-products.

Treatment	Sensory Attributes				
	Color Intensity	Off Colors	Marbled	Aroma Intensity	Off Aromas
1	72 ± 15	1 ± 2	64 ± 16	67 ± 17	3 ± 4
2	73 ± 14	1 ± 2	66 ± 16	71 ± 11	3 ± 4
3	74 ± 15	0 ± 1	67 ± 17	73 ± 17	3 ± 4
4	67 ± 18	1 ± 3	67 ± 16	72 ± 11	3 ± 5
5	69 ± 19	1 ± 3	63 ± 19	74 ± 13	3 ± 6

Data are expressed as means ± SD. 1—Control; 2—*S*. *equorum* S2M7; 3—*L*. *sakei* CV3C2; 4—*S*. *equorum* S2M7/*L*. *sakei* CV3C2; 5—*S*. *equorum* S2M7/*L*. *sakei* CV3C2/yeast 2RB4. In the same column, different letters represent significantly different means (*p* < 0.05).

## Data Availability

Data is contained within the article. Further details are avavailable on request from the corresponding author.
